# Breast Cancer Subtype Specific Classifiers of Response to Neoadjuvant Chemotherapy Do Not Outperform Classifiers Trained on All Subtypes

**DOI:** 10.1371/journal.pone.0088551

**Published:** 2014-02-18

**Authors:** Jorma J. de Ronde, Marc Jan Bonder, Esther H. Lips, Sjoerd Rodenhuis, Lodewyk F. A. Wessels

**Affiliations:** 1 Department of Molecular Carcinogenesis, The Netherlands Cancer Institute, Amsterdam, The Netherlands; 2 Department of Molecular Pathology, The Netherlands Cancer Institute, Amsterdam, The Netherlands; 3 Department of Pathology, The Netherlands Cancer Institute, Amsterdam, The Netherlands; 4 Department of Medical Oncology, The Netherlands Cancer Institute, Amsterdam, The Netherlands; 5 Faculty of EEMCS, Delft University of Technology, Delft, The Netherlands; National Cancer Center, Japan

## Abstract

**Introduction:**

Despite continuous efforts, not a single predictor of breast cancer chemotherapy resistance has made it into the clinic yet. However, it has become clear in recent years that breast cancer is a collection of molecularly distinct diseases. With ever increasing amounts of breast cancer data becoming available, we set out to study if gene expression based predictors of chemotherapy resistance that are specific for breast cancer subtypes can improve upon the performance of generic predictors.

**Methods:**

We trained predictors of resistance that were specific for a subtype and generic predictors that were not specific for a particular subtype, i.e. trained on all subtypes simultaneously. Through a rigorous double-loop cross-validation we compared the performance of these two types of predictors on the different subtypes on a large set of tumors all profiled on the same expression platform (n = 394). We evaluated predictors based on either mRNA gene expression or clinical features.

**Results:**

For HER2+, ER− breast cancer, subtype specific predictor based on clinical features outperformed the generic, non-specific predictor. This can be explained by the fact that the generic predictor included HER2 and ER status, features that are predictive over the whole set, but not within this subtype. In all other scenarios the generic predictors outperformed the subtype specific predictors or showed equal performance.

**Conclusions:**

Since it depends on the specific context which type of predictor – subtype specific or generic- performed better, it is highly recommended to evaluate both specific and generic predictors when attempting to predict treatment response in breast cancer.

## Introduction

Breast cancer is a heterogeneous disease, which can be subdivided into distinct subtypes. Based on clinical features, including age, tumor grade, and TNM stage, patients can be stratified into more homogeneous subgroups. By measuring estrogen receptor (ER), progesterone receptor (PR) and human epidermal growth factor receptor 2 (HER2) expression, we can stratify tumors into a HER2 positive set (HER2+, either ER+ or ER−), a Luminal set (HER2−, ER+) and a Triple Negative set (HER2−, ER−, PR−; TN). These three different subtypes of breast cancer are considered distinct diseases and are approached accordingly in the clinic. Breast cancer patients of all subtypes receive chemotherapy as part of the treatment process.

Neoadjuvant therapy, the administration of therapeutic agents before the main treatment (typically surgery), reduces the mortality of breast cancer patients [1]. However, some patients only experience the downside of the therapy (i.e. toxicity) and not the benefit (i.e. increased survival). It has been shown that patients who achieve a pathological complete response after neoadjuvant treatment have a higher chance of relapse free survival [2], [3]. Because neoadjuvant treatments can be very toxic and not all patients benefit from the treatment, it would be desirable if the non-responders to therapy can be accurately separated from the responders. Multiple approaches to predict the response of patients to neoadjuvant treatment have been undertaken [4]–[10], a number of which were reviewed in [11]. Unfortunately, up to now none of the published predictors have been applied in the clinical setting. This lack of clinical implementation can be attributed to the fact that the results that were reported in the original papers are either not reproducible in external data sets or the fact that the reported accuracy is not high enough to be used in clinical decision making.

The best performing predictor of chemotherapy response published to date has an area under the receiver operator curve (AUC) of 0.805 [8]. In a subsequent independent validation study, AUCs of 0.711 (T-FAC treatment) and 0.584 (FAC treatment) were achieved for this same predictor [12]. While this is an improvement over previous attempts, the accuracy of this predictor is insufficient for clinical decision making. In order for a predictor to be useful in the clinic, it should have a high true positive rate (sensitivity) and a high true negative rate (specificity). How high ‘high’ should be depends on the clinical decision to be made. For example, if a predictor is employed to predict whether a chemotherapeutic treatment will benefit a specific patient, the decision will have far reaching consequences. However, if no alternative methods of predicting response to treatment are available, any sensitivity and specificity rates better than random will be useful. While it is very hard to set a specific required minimal specificity and sensitivity rate, we believe at least 0.9 should be required for both (note that the specificity and sensitivity determine the AUC). This predictor was trained on all available samples in the training dataset, irrespective of breast cancer subtype.

In this study, we set out to determine if we could increase the accuracy of the chemotherapy response predictors by creating predictors that are specific for each breast cancer subtype, instead of a predictor that was trained on all subtypes combined. In order to make sure that our results would not be biased towards a certain type of methodology we included six feature selection approaches and six classifiers. These methods reflect commonly used methods in the context of class prediction. Additionally, to answer the question of how generally applicable our results are, we included predictors based on gene expression data and predictors based on clinical features.

## Methods

### Samples

For this study we selected three gene expression data sets, all hybridized on either the Affymetrix HG U133A platform or the Affymetrix HG U133 plus 2.0 platform. The three sets consisted of the microarray quality control II (MAQC2) breast cancer dataset [13]; a breast cancer dataset from the Department of Breast and Endocrine Surgery of the Osaka University (BESOU) [14] and a breast cancer dataset from the MD Anderson Cancer Center (MDACC) [12]. For all three datasets, informed consent from the included patients and approval from an ethics committee were obtained (the University of Texas M.D. Anderson Cancer Center ethical committee for the MAQC2 study, the Ethics Review Committee at Osaka University Hospital for the BESOU dataset, and the institutional review boards of each participating institution for the MDACC study). These datasets are publicly available from the Gene Expression Omnibus website (MAQC2 GEO ID is GSE16716, BESOU GEO ID is GSE32646, and MDACC GEO ID is GSE20271). From these sets we extracted the samples that were treated with taxol followed by 5-fluorouracil, (Adriamycin or Epirubicin), cyclophosphamide (T-FAC or T-FEC). Patients that were treated with a regimen including trastuzumab or FAC alone were removed from our dataset in order to get a more homogeneous treatment group. After this selection the dataset consisted of 394 samples in total. Unfortunately not all of these had all clinical data available, so for analyses based on clinical features the total dataset consisted of 374 samples. Table 1 and Table S1 show an overview of the characteristics of this set of patients.

**Table 1:** Distribution of samples in the subgroups.

Stratification	pCR (%)	No pCR (%)
**Clinical subtypes**		
**HER2 positive**	31 (38)	51 (62)
**Luminal**	14 (9)	185 (91)
**Triple Negative**	42(37)	71 (63)
**HER2 positive, ER negative**	25 (56)	20 (44)
**HER2 positive, ER positive** *	6 (16)	31 (84)
**No stratification**		
**All**	87 (22)	307 (78)

The sample sizes that are depicted are from the expression based predictors. The sample sizes for the clinical predictors are a bit lower due to missing data and can be found in Table S1.

*The HER2 positive, ER positive group was not included in the analysis due to the small sample size.

In these datasets, a pathological complete response (pCR) was defined as no residual invasive cancer in the breast and axillary lymph nodes. Samples were defined to be ER-positive or PR-positive when 10% or more of the tumor cells showed positive staining of ER or PR respectively, based on immunohistochemistry. Samples were marked as positive for HER2 when there was strong membrane staining (3+) or the sample had a gene copy number equal to or greater than 2.0 as measured by Fluorescent In Situ Hybridization (FISH ratio greater than 2.0 for the BESOU dataset).

### Data preparation

All datasets (raw data) were downloaded from GEO [15]. The samples were background corrected and normalized using GeneChip-RMA [16] and subsequently log2 transformed. Since we combined datasets which originated from different experiments and platforms we applied a normalization strategy to enable reliable combination of the different datasets. First we verified the individual probe quality by employing ProbeMapper [17]. We selected the probes that were mapped to the same transcript by the vendor, the bioconductor annotation packages and a BLAST mapping of the probes to the set of transcripts of the latest human genome build (Hg19). In addition, we discarded the probes that were mapped to multiple genes. The probes that passed these tests were median centered, i.e. we set the median expression of each probe per dataset to match the median expression of that probe in the MAQC2 dataset. The median centering was performed by taking into consideration the ratio of clinical subtypes and response rates in each dataset, i.e. the median expression of a gene within a dataset was determined on a subset that was chosen such that it matched the other datasets with respect to the percentages of clinical subtypes and response rates. We chose the largest possible subsets that satisfied these constraints. For the subsequent analysis we selected the most informative probe per gene, i.e. the probe which showed the highest standard deviation across all data sets.

### Feature selection and classification

After probe matching and gene median centering, 12010 genes were entered into the subsequent analysis. In addition to expression based predictors we also investigated the predictive power of clinical features. For these predictors we selected ER status, PR status, HER2 status, TNM-stage, age and grade as the features to be used. All of these are routinely determined in the clinic and were directly available in the datasets we selected.

We employed three feature selection approaches for the expression based predictors and three for the clinical predictors. We chose different feature selection approaches for the expression and the clinically based predictors, since the expression features are all continuous valued and most of the clinical features are categorical, implying different requirements for feature selection. The feature selection approaches employed by the expression predictors were: Wilcoxon-Mann-Whitney test (WMW), Wilcoxon-Mann-Whitney test after removal of features with a Pearson correlation exceeding 0.75 (WMW-uncor.) and the ratio of between- to within group sum of squares (BWR). These methods are either available in R [18] or were implemented in R. The ranking approaches employed for the clinical features were: information gain (Inf.gain), correlation feature selection (CFS) and RELIEF. These feature selection methods are available in the “FSelector” R package [19]. The features were ranked by their respective scores when comparing responder samples to non-respond samples (i.e. their discriminative power) in order to select the best features for classification.

The classifiers that we employed were the same for the expression data and the clinical data. We tested the following classifiers: the J48 decision tree (J48), the 3-nearest neighbor classifier (3NN) based on Euclidean space, the nearest mean classifier (in Euclidean space), logistic regression (LREG), naive Bayes (NB) and a support vector machine (SVM) (WeKa SMO with default settings). This represents a wide range of classifiers including linear and non-linear classifiers as well as classifiers designed for discrete and continuous features. All classifiers were implemented in the “RWeka” R package [20], [21].

We implemented each possible combination of classifier and feature ranking approach, except for the J48 classifier since this classifier includes its own feature selection approach. This resulted in 16 clinical and 16 expression based predictors. We will refer to a specific combination of a set of selected features and the associated classifier as a predictor.

### Estimation of predictive performance

**Figure 1:**
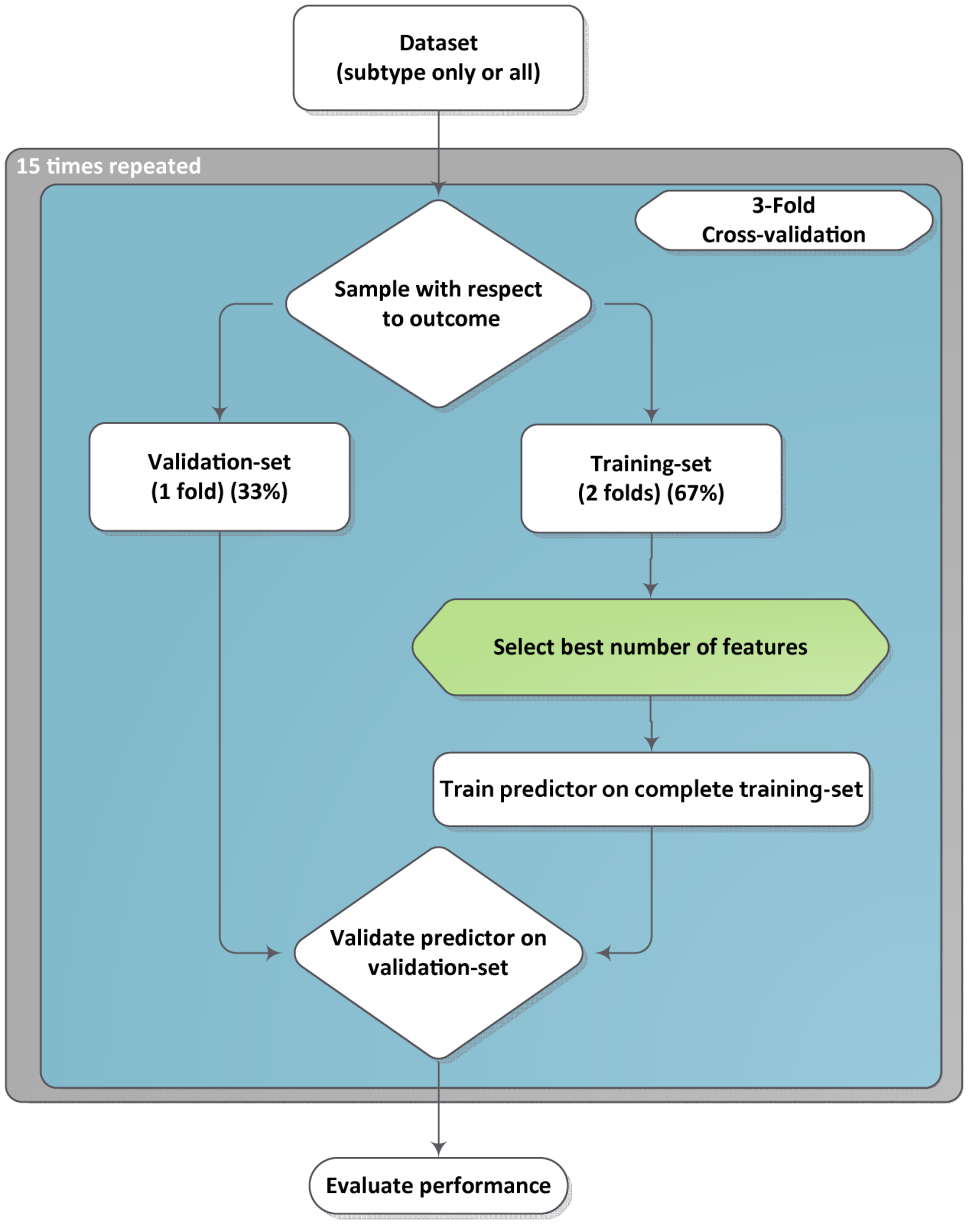
Cartoon of the double loop cross-validation scheme. Our analysis employed a double look cross-validation. The inner loop determines the optimal number of features to be used by a specific combination of feature selection and classifier, here depicted by the green block. This inner loop uses 2/3 of all data (i.e. the training data), the remaining 1/3 is employed to measure the performance of the trained classifier (i.e. a 3 fold cross-validation setup). The outer loop is repeated 15 times in order to get an average AUC for each predictor.

To estimate the accuracy of the predictors we employed the AUC. The AUC is estimated through a double loop cross-validation strategy, illustrated in Figure 1.

Full details of the procedure are outlined in the supplementary materials and methods (Methods S1 and Figure S1), in summary the following steps are taken: 1) the input dataset is divided into 2/3 for the training set and 1/3 for the validation set; 2) the training set (i.e. 2/3 of all data) is employed to determine the optimal number of features to be used in each feature selection method and classifier combination (i.e. for each of the 16 possible combinations of feature selection method and classifier, a single, optimal number of features is determined); 3) the training set is then used to train each of the 16 predictors with the number of features previously determined to be optimal for each combination; 4) finally the performance of each feature selection and classifier combination is assessed by applying these 16 predictors to the validation set (i.e. 1/3 of all data). The split into training and validation sets was performed such that the ratios of subtypes and response rates within the two sets were equal. This whole procedure was repeated 15 times in order to get a more accurate average performance per predictor and subtype. This double loop cross-validation procedure is similar to the approach that was employed by Popovici et al. [13] and Wessels et al. [22].

For the non-subtype specific predictors, we divided all data of all subtypes into three equal parts, i.e. the dataset was subdivided into 2/3 of the samples in the training set and the remaining 1/3 in the validation set. For the subtype specific predictors the input dataset consisted only of samples of the specific subtype being analyzed (i.e. the 2/3 training set and 1/3 validation set consisted only of samples belonging to the relevant subtype). In order to compare the performance of the non-subtype specific predictor with the subtype specific predictor on a given subtype, say TN, the non-subtype specific predictor was trained on a training set consisting of *all* subtypes, but only validated on a the validation set consisting of TN samples.

Finally we selected the best performing predictor per subtype for both the subtype specific and the non-subtype specific predictors in order to compare their performances. To compare the AUC values of non-subtype specific predictors to the AUC values of subtype specific predictors we employed the two-sided t-test.

## Results

### Subtype specific versus non-subtype specific predictors

We employed a stratification based on ER and HER2 status. We stratified patients into a HER2 positive group (HER2+), a Luminal group (HER2−, ER+) and a Triple Negative group (HER2−, ER−)(TN). We further subdivided the HER2-positive group based on ER status, resulting in a HER2-positive and ER-negative group (HER2+, ER−) and a HER2-positive and ER-positive group (HER2+, ER+). Unfortunately, the (HER2+, ER+)-group contained too few samples to analyze this group separately with adequate power (n = 37).

**Figure 2:**
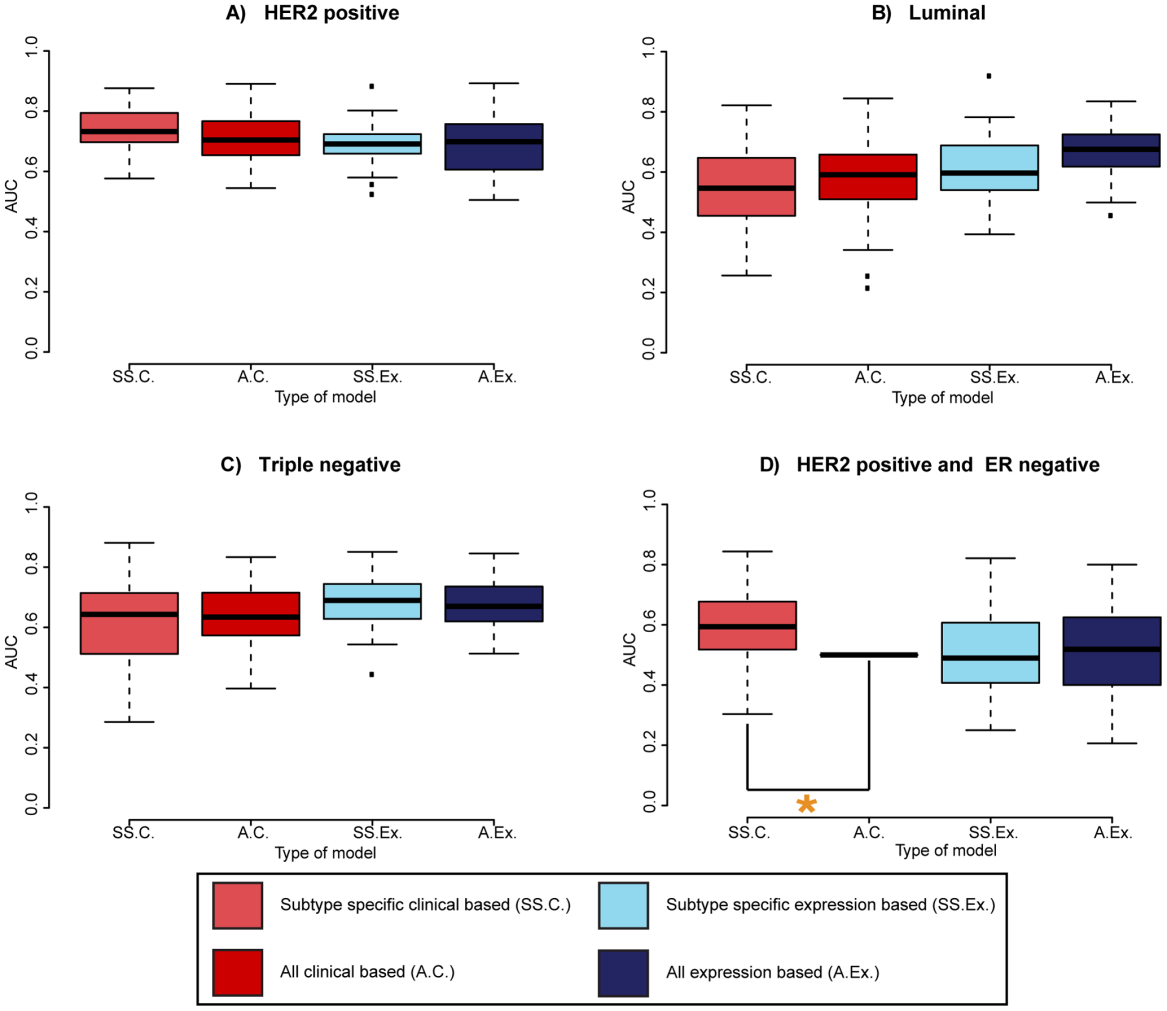
The AUC scores for the best performing predictors on each subtype. AUCs for the (A) HER2 positive subtype; (B) Luminal subtype; (C) Triple negative subtype and (D) HER2 positive and ER negative subtype. The red bars represent the clinical predictors, blue bars the expression based predictors and darker colors represent non-subtype specific predictors. When two boxplots are connected with a u-shaped line, the means of the AUC distributions are significantly different for the experiment represented by the boxplots (two-sided t-test, p<0.05, Bonferroni multiple testing corrected.)

Figure 2 shows the comparison of the best performing classifiers for the subtype specific and non-subtype specific predictors.

#### Predictors based on clinical features

The AUCs for the clinical subtype specific and non-subtype specific predictors were highly similar for most sets (HER2+, TN and ER+) (Red boxplots in Figure 2). Only in the HER2+, ER− subgroup there was a significant difference, where the subtype specific predictor had a significantly higher AUC than the non-subtype specific (multiple testing corrected p-value: 4.3×10^−5^). None of the other groups showed a significant difference in AUC between the subtype specific and non-subtype specific clinical predictors.

#### Predictors based on gene expression data

The AUC comparisons for the expression based predictors (blue boxplots in Figure 2) mostly mirrored the results for the clinical features based predictors. All groups showed highly similar results for the subtype specific and the non-subtype specific predictors. Only in the Luminal group, a borderline significant difference in AUC between the subtype specific and non-subtype specific predictor was observed (corrected p-value: 0.0709).

Table 2 shows the best performing feature selection method-classifier combination for each dataset (these correspond to predictors whose AUCs were compared in Figure 2). The exact average AUC scores and details about the different data sets can be found in Table S2.

**Table 2:** Characteristics of the optimal predictors for the different subtypes.

	Clinical		Gene Expression	
Stratification	Subtype specific	Non specific	Subtype specific	Non specific
**Luminal**	LREG-Relief	NM-Relief	NM-WMW	NB-BWR
**Triple Negative**	NM- CFS	NB-Relief	NB-BWR	LREG-WMW- uncor.
**HER2-positive**	NB-Relief	NB-CFS	NB-WMW	NB-WMW
**HER2-positive, ER-negative**	3NN-CFS	NM-CFS	NB-WMW	LREG-BWR

In each cell the optimal combination of classifier, and feature selection method, is shown.

**Legend:** **classifiers:** NB = Naive Bayes, NM = Nearest Mean, LREG: Logistic regression, SVM = Support vector machine, 3NN = 3-Nearest Neighbor; **Feature selection methods:** CFS = Correlated feature selection, WMW = Wilcoxon-Mann-Whitney, BWR = Ratio between to within class sum of squares, WMW-uncor. = Wilcoxon-Mann-Whitney where correlated features are removed, Inf.gain = information gain.

## Discussion

From our analysis we can conclude that there is very little difference in performance between building subtype specific predictors compared to building non-subtype specific predictors (i.e. combining data of all subtypes and training on that dataset). This is true for the predictors employing clinical features as well as the predictors employing gene expression predictors. We did observe that in the Luminal subgroup all the non-subtype specific models (gene expression or clinical features based) tended to achieve better AUC scores compared to the subtype specific predictors, although this difference was not significant. The Luminal group has been reported to consist of a combination of two distinct intrinsic subtypes, Luminal A and Luminal B. This further subdivision of the Luminal subtype could prove interesting, since these subtypes were reported to show different response rates following neoadjuvant treatment. Table S3, Table S4, and Figure S2 show the results of our analysis of the Luminal intrinsic subtypes, which were classified according to the “PAM50” predictor [23]. In the expression based model, we observed a significant difference between the subtype specific predictor and the non-subtype specific predictor in the Luminal A and Luminal B subgroups. Analogous to the Luminal subgroup analysis, the non-subtype specific predictors outperformed the subtype specific predictors. This could be explained by the fact that positive events (i.e. pathological complete responses) are rare in these sets (13, 13, and 14 cases, corresponding to 9%, 13%, and 14% of the samples showed a pCR in the Luminal A, Luminal B, and Luminal groups, respectively). Having few samples in the minority class will make it difficult to train a predictor that correctly classifies samples from this minority class, leading to lower AUC values. The higher performance of the non-subtype specific predictor (compared to the subtype specific predictor) could be explained by the fact the complete dataset on which it was trained has a higher (absolute) number of samples in the minority class (i.e. responders), which in turn might lead to better classification of samples in the minority class in the validation set and a higher AUC as a result.

For the TN and HER2+ subtypes, there was no clear difference between the subtype specific and the non-subtype specific predictors. Only for the HER2-positive and ER-negative subgroup, we found a significant difference in clinical feature based predictor performance. The subtype specific predictor, based on clinical features, was superior to the non-subtype specific model. While the AUC of the subtype specific model was 0.59, the AUC of the non-subtype specific model was exactly 0.5. This could be explained by the fact that in most iterations of the predictor training, the non-subtype specific predictor included only a single feature. This single feature was ER status (in some cases combined with either PR or HER2). Taken over the whole dataset, ER status (or PR and HER2) is highly predictive of response to chemotherapy. However, the HER2+, ER− subtype is, by definition, completely ER negative and HER2 positive, so the ER and HER2+ status feature will not yield any predictive power in this subgroup. The subtype specific predictor did not include ER or HER2 status, but included age, stage and grade. Given that the subtype specific predictor included more informative features, it was no surprise that it could achieve higher AUC values. This scenario forms a clear example of when a subtype specific predictor is preferred, i.e. a feature is predictive over the whole dataset, but is non-informative within a specific subgroup of that dataset. When we look at the same subtype, but now employing gene expression data, the difference between the subtype specific and non-subtype specific predictors, however. The selected features in that classifier are predictive over both the entire set and this specific subgroup of samples.

In our analysis we did not take into account that there is a large difference in the size of the training set between subtype specific and non-subtype specific predictors. That is, we compared the subtype specific predictors, the predictors based only on data from one subtype, to a predictor that was trained on data from all subtypes. This means that the training set sizes of the non-subtype specific predictors were two to four times as large as the subtype specific training sets (depending on the size of the subtype relative to the whole dataset). These training set size differences have an influence on predictor performance and performance estimation. Since we focused on comparing subtype specific to non-subtype specific predictors in real world situations, we opted to compare performance in this manner. When a dataset is to be analyzed, one would always want to use as much data as available so it would not make sense to only take part of the combined dataset purely for comparison reasons. Our results suggest that the larger training set of the generic predictor outweighs the benefits of a subtype specific training set. This in turn implies the important notion that there are features in the data that can predict response to therapy that are shared amongst different subtypes. That is, if there was no commonality between predictive factors in the different subtypes, the increased training set size would not increase the performance of the generic predictor. It has been shown that patients benefit from receiving subtype specific treatments instead of a general treatment [24], [25]. When patients receive treatment that is specific for a particular subtype, a predictor that combines all data will not only have to deal with heterogeneity in subtypes, but also in treatment regimens received. It remains to be seen if a non-subtype specific predictor will, in such a scenario, still perform as well as a predictor that was trained for a specific treatment and subtype.

In this study we combined publicly available datasets in order to have as many samples as possible available for analysis. Since treatment and microarray platform could potentially be major confounders in an analysis such as the one we present here, we opted to limit our dataset to samples from patients receiving similar treatment and which were analyzed on the same microarray platform. To account for institute specific confounders (and consequently also genetic background confounders to a degree, given that one institute is located in Japan and two institutes procured samples from US and Europe), we normalized our datasets by median centering per probe, per institute. Even with these stringent criteria and normalization procedure, confounding effects could not be ruled out. Until larger homogeneous datasets become available, such potential confounders will remain an issue.

In recent papers studying outcome prediction after T-FAC or T-FEC and FAC or AC neoadjuvant therapy, no predictors specific for breast cancer subtypes were built [4], [8], [9], [13]. Therefore, we cannot directly assess if the subtype specific predictors that we built perform better or worse than previously published predictors. However, we could compare the non-subtype specific predictors (i.e. built and tested on all subtypes at once). More specifically, we compared the performance of our predictors to the predictors published alongside the three Affymetrix datasets which we included in our study. Unfortunately, the paper that was published alongside the BESOU data did not include AUC values so we could not compare their performance to ours. Instead, the authors of this paper [9], focused on finding the best model to achieve a high negative predictive value instead of a high AUC. In the paper accompanying the MAQC2 dataset [13], the authors presented an expression based predictor that combined LREG with BWR. The gene expression based predictor published with the MDACC dataset [12], was a predictor based on DLDA (Naive Bayes (NB)), which included 30 genes, selected based on the t-test p-value. The average AUC of the Popovici et al. model was: 0.805, Tabchy et al. reported an AUC 0.711 for the T-FAC treated samples. Our highest average AUC value was 0.768 (NB with BWR using 30 features). Given the variance we observed around our AUC estimate, these AUC values are most likely not statistically significantly different.

Tabchy et al. also validated a nomogram based on clinical data, which achieved an AUC of 0.89 for the T-FAC treatment arms. Our best clinical feature predictor was the predictor employing NB and relief using six features, which achieved an average AUC of 0.796. Again, the variance of the double loop CV estimate is of such a magnitude that these values are most likely not significantly different.

A possible explanation for the slightly lower AUC values for our predictors could lie in the fact that we classified more samples and -potentially- our AUC estimates are more accurate due to our larger sample set. We included 2.9 times as many samples compared to Tabchy et al. and 1.7 times as many samples compared to Popovici et al. In addition, we combined data from multiple sets and even though the patient characteristics were not significantly different, this could have an influence on the AUC. Differences in estimated AUC values could also be attributed to the training and validation procedures employed. To test this we applied our double-loop cross validation on the Tabchy and Popovici datasets. Our best performing gene expression based predictors showed AUCs of 0.807 and 0.780 respectively, which is very close to the reported performances. This indicates that the published performances are robust, but dataset specific. As we trained and tested on a larger dataset, and as the training and testing procedure has been proven to be robust, our achieved AUC of 0.768 is probably closer to the real value. However, as indicated above, these values are within the error margins of the experiments and since we compared the relative performance of specific to generic predictors, this performance difference has no consequences for the results presented here.

## Conclusions

In general, subtype specific predictors would be preferred over non-subtype specific predictors. However, when the phenotype to be predicted has a skewed distribution, like response in the Luminal subtype, a non-subtype specific predictor (i.e. based on all data combined) will outperform the subtype specific predictor. This implies that there are features that are predictive of response to chemotherapy that are shared amongst different subtypes and resistance mechanisms are not exclusive to particular subtypes. In specific cases where features are predictive over the whole dataset, but uninformative within a specific subgroup, like ER and HER status in the HER2-positive and ER-negative subgroup, a subtype specific predictor will offer a significant performance gain over non-subtype specific predictors.

For breast cancer specifically - with the exception of the HER2-positive, ER-negative group -, we can conclude that building a subtype specific predictor offers equal performance compared to a predictor based on all available data. However, when it is unknown which of the scenarios mentioned above is present in the data, it would be advised to analyze both subtype specific and non-subtype specific predictors.

## Supporting Information

Methods S1 (DOCX)

Figure S1 Illustration of the double loop cross-validation scheme. Legend: 3FCV: 3 fold cross-validation, CFS: Correlated feature selection method, Inf.gain = information gain, WMW = Wilcoxon-Mann-Whitney test, WMW-uncor. = Wilcoxon-Mann-Whitney with correlated features removed, BWR = between to within group sum of squares. NMC =  nearest mean classifier, LREG = logistic regression, NB = naïve Bayes, 3NN = k-nearest neighbor, SVM = support vector machine.
(TIF)

Figure S2 AUCs for the (A) Luminal A subtype; (B) Luminal B subtype. The red bars represent the clinical predictors, blue bars the expression based predictors and darker colors represent non-subtype specific predictors. When two boxplots are connected with a u-shaped line, the means of the AUC distributions are significantly different for the experiment represented by the boxplots (two-sided t-test, p<0.05, Bonferroni multiple testing corrected.)
(TIF)

Table S1 Basic description of the dataset used to analyze the influence of subtype specific versus the non-subtype specific models for predicting outcome after treatment. There is a small difference in samples size for the clinical versus the expression based model as there are some samples which lack description of clinical features.
(XLSX)

Table S2 AUC values for the models that were included in the analysis. Each tab of the excel file describes a different set of models. The different models are in the rows and the different subtypes are next to each other.
(XLSX)

Table S3 Distribution of samples in the Luminal A and Luminal B subtypes. The sample sizes shown are the sample sizes as employed by the expression based predictors. The sample sizes of the clinical predictors were a bit lower due to missing data and can be found in Table S1.
(DOCX)

Table S4 Characteristics of the optimal predictors for the different subtypes. In each cell the optimal combination of classifier, and feature selection method, is shown. **Legend: classifiers:** NB = Naive Bayes, NM = Nearest Mean, LREG: Logistic regression, SVM = Support vector machine, 3NN = 3-Nearest Neighbor; **Feature selection methods:** CFS = Correlated feature selection, WMW = Wilcoxon-Mann-Whitney, BWR = Ratio between to within class sum of squares, WMW-uncor. = Wilcoxon-Mann-Whitney where correlated features are removed, Inf.gain = information gain.
(DOCX)
