# Supplementary material for: Breast Cancer Subtype Specific Classifiers of Response to Neoadjuvant Chemotherapy Do Not Outperform Classifiers Trained on All Subtypes
Source: PLoS One. 2014 Feb 18;9(2):e88551. doi: 10.1371/journal.pone.0088551 (PMC3928239; doi:10.1371/journal.pone.0088551)
Supplement: Methods S1 — (DOCX) [file pone.0088551.s001.docx]

## Supplemental methods S1

### Estimation of predictive performance

To estimate the accuracy of the predictors we employed the area under the receiver operator curve (AUC). The AUC is estimated through a double loop cross-validation strategy.

Figure 1 illustrates the experimental design. In the inner-loop, marked in green (Figure 1), the optimal number of features is selected per classifier (C) and feature selection (FS) combination, i.e. per predictor. In the outer-loop, marked in blue (Figure 1), all training samples were used to train the predictor, employing the number of features per predictor that was determined in the previous steps. This classifier was then evaluated by measuring the predictive accuracy on the samples that were not used in either the training of the classifier or in selecting the optimal number of features. In both the inner loop and in the outer loop, a three-fold cross-validation (3FCV) was used. In the outer-loop the 3FCV is repeated 15 times and the 3FCV of the inner-loop was repeated 5 times per outer-loop iteration. We chose to use fewer iterations in the inner as compared to the outer loop as this had a large influence on the runtime and we reasoned that the confidence intervals of the inner loop did not require a high accuracy since we were only interested in the maximum average AUC to select the best number of features. The stratifications of the data into a training and an evaluation set were performed with respect to response rates.

We calculated the optimal number of features to be used for each predictor by averaging the best performing number of features selected in each inner loop iteration, per FS-C combination. To limit the number of tests to be performed, we fixed the number of features that could be used. For the clinical predictors this ranged from one to six features in steps of one and for the gene expression data the number of features ranged from 10 to 120 features in steps of 10.

To calculate the AUC, we evaluated the class posteriors returned by the predictors. The AUC calculation itself was performed using the “ROCR” R package[[1](#_ENREF_1)]. By averaging the AUCs for the three folds repeated either 15 (outer) or 5 (inner) times, we could estimate the AUC performance of a new set including the corresponding confidence intervals.

The double loop cross-validation procedure is similar to the approach that was employed by Popovici et al.[[2](#_ENREF_2)] and Wessels et al.[[3](#_ENREF_3)].

### Luminal A and B

The Luminal subgroup can be subdivided into two more homogeneous groups, Luminal A and Luminal B. This classification can potentially be interesting, since these subtypes have been observed to show a different outcome rate after neoadjuvant treatment. We set out to analyze whether a subtype specific classifier delivers a performance gain in these two groups. In order to assign the samples to the correct intrinsic subtype, we employed the “PAM50” predictor[[4](#_ENREF_4)]. Next, we selected the samples that were assigned to Luminal A or Luminal B. Table S2 shows the distribution of the samples over these two subtypes. Note that Luminal A and Luminal B subtypes are not restricted to samples assigned to the Luminal class (classification based on immunohistochemistry), which resulted in a different (i.e. larger) number of samples in the Luminal A and Luminal B subtypes combined, compared to the Luminal (IHC) subtype (i.e. the “PAM50” subtypes are derived independently from the clinical subtypes).

For the Luminal A and Luminal B samples we employed the same analyses as for the clinical subtypes. We employed both the gene based methods and the clinical feature based methods. Figure S2 shows the performance of the best performing classifiers for the subtype specific and non-subtype specific predictors.

In both the Luminal A and B group there was a significant differences between the subtype specific and non-subtype specific predictors (corrected p‐values: Luminal A: 2.517 10^‐4,^, Luminal B: 3.765 10^-24^). In both cases the non-subtype specific models outperformed the subtype specific models. For the clinical based models, no differences were observed. The predictors for the best performing models are shown in Table S3, more detailed results are shown in in Table S1.

# References

1. Sing T, Sander O, Beerenwinkel N, Lengauer T (2009) ROCR: Visualizing the performance of scoring classifiers.

2. Popovici V, Chen W, Gallas BG, Hatzis C, Shi W, et al. (2010) Effect of training-sample size and classification difficulty on the accuracy of genomic predictors. Breast cancer research : BCR 12: R5.

3. Wessels LF, Reinders MJ, Hart AA, Veenman CJ, Dai H, et al. (2005) A protocol for building and evaluating predictors of disease state based on microarray data. Bioinformatics 21: 3755-3762.

4. Parker JS, Mullins M, Cheang MC, Leung S, Voduc D, et al. (2009) Supervised risk predictor of breast cancer based on intrinsic subtypes. Journal of clinical oncology : official journal of the American Society of Clinical Oncology 27: 1160-1167.
