# Supplementary figures and images for: Breast Cancer Subtype Specific Classifiers of Response to Neoadjuvant Chemotherapy Do Not Outperform Classifiers Trained on All Subtypes
Source: PLoS One. 2014 Feb 18;9(2):e88551. doi: 10.1371/journal.pone.0088551 (PMC3928239; doi:10.1371/journal.pone.0088551)

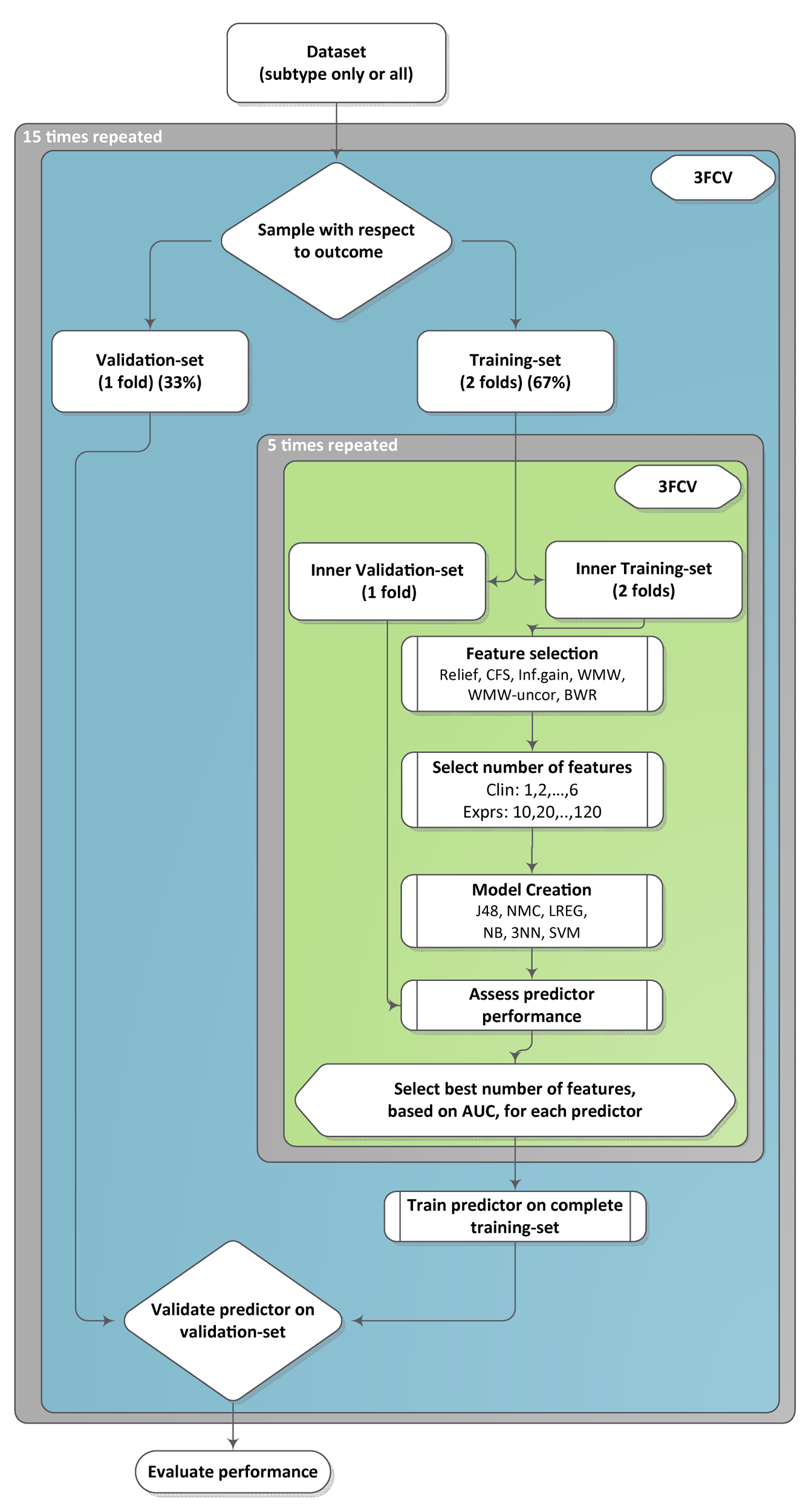

Supplement: Figure S1 — Illustration of the double loop cross-validation scheme. Legend: 3FCV: 3 fold cross-validation, CFS: Correlated feature selection method, Inf.gain = information gain, WMW = Wilcoxon-Mann-Whitney test, WMW-uncor. = Wilcoxon-Mann-Whitney with correlated features removed, BWR = between to within group sum of squares. NMC = nearest mean classifier, LREG = logistic regression, NB = naïve Bayes, 3NN = k-nearest neighbor, SVM = support vector machine. (TIF) [file pone.0088551.s002.tif]

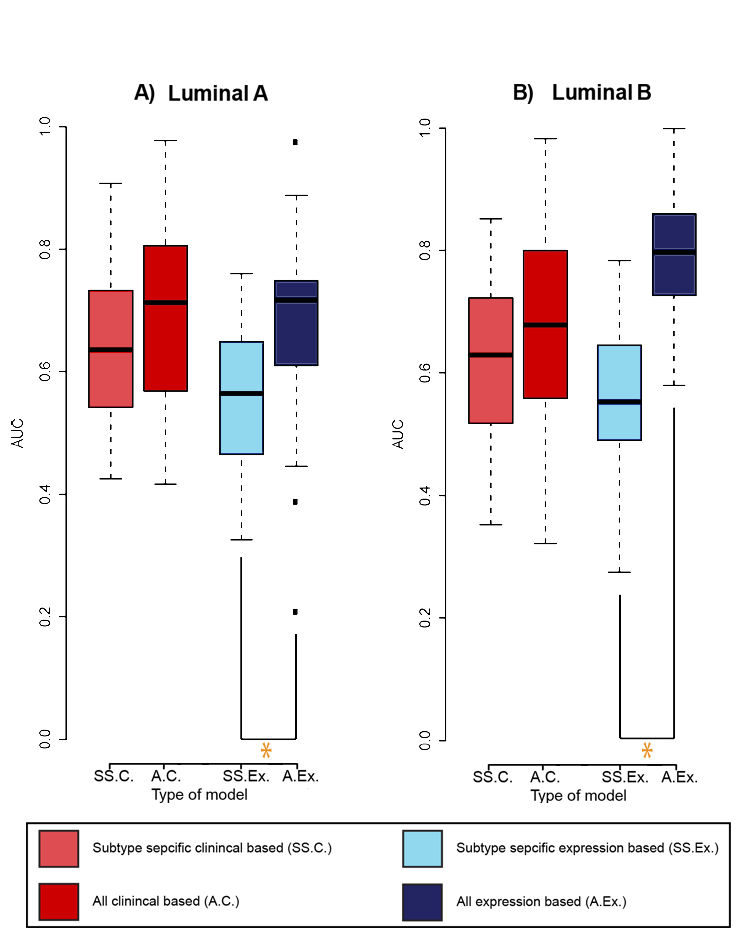

Supplement: Figure S2 — AUCs for the (A) Luminal A subtype; (B) Luminal B subtype. The red bars represent the clinical predictors, blue bars the expression based predictors and darker colors represent non-subtype specific predictors. When two boxplots are connected with a u-shaped line, the means of the AUC distributions are significantly different for the experiment represented by the boxplots (two-sided t-test, p<0.05, Bonferroni multiple testing corrected.) (TIF) [file pone.0088551.s003.tif]
