# Supplementary material for: Breast Cancer Subtype Specific Classifiers of Response to Neoadjuvant Chemotherapy Do Not Outperform Classifiers Trained on All Subtypes
Source: PLoS One. 2014 Feb 18;9(2):e88551. doi: 10.1371/journal.pone.0088551 (PMC3928239; doi:10.1371/journal.pone.0088551)
Supplement: Table S3 — Distribution of samples in the Luminal A and Luminal B subtypes. The sample sizes shown are the sample sizes as employed by the expression based predictors. The sample sizes of the clinical predictors were a bit lower due to missing data and can be found in Table S1. (DOCX) [file pone.0088551.s006.docx]

| **Stratification** | **pCR (%)** | **No pCR (%)** |
| --- | --- | --- |
| **Intrinsic subtypes** |  |  |
| **Luminal A** | 13 (9) | 136 (91) |
| **Luminal B** | 13 (13) | 85 (87) |
