# Supplementary material for: Breast Cancer Subtype Specific Classifiers of Response to Neoadjuvant Chemotherapy Do Not Outperform Classifiers Trained on All Subtypes
Source: PLoS One. 2014 Feb 18;9(2):e88551. doi: 10.1371/journal.pone.0088551 (PMC3928239; doi:10.1371/journal.pone.0088551)
Supplement: Table S4 — Characteristics of the optimal predictors for the different subtypes. In each cell the optimal combination of classifier, and feature selection method, is shown. Legend: classifiers: NB = Naive Bayes, NM = Nearest Mean, LREG: Logistic regression, SVM = Support vector machine, 3NN = 3-Nearest Neighbor; Feature selection methods: CFS = Correlated feature selection, WMW = Wilcoxon-Mann-Whitney, BWR = Ratio between to within class sum of squares, WMW-uncor. = Wilcoxon-Mann-Whitney where correlated features are removed, Inf.gain = information gain. (DOCX) [file pone.0088551.s007.docx]

|  | **Clinical** | | | **Gene Expression** | |
| --- | --- | --- | --- | --- | --- |
| **Stratification** | Subtype specific | Non specific | Subtype specific | | Non specific |
| **Luminal A** | NB-Inf.gain | NM-Relief | LREG-WMW-uncor. | | NB-WMW |
| **Luminal B** | NM-CFS | NM-Relief | NB-WNW-uncor. | | SVM-BWR |
